# Mapping of *cis*-acting expression quantitative trait loci in human scalp hair follicles

**DOI:** 10.1186/s12895-020-00113-y

**Published:** 2020-11-10

**Authors:** Marisol Herrera-Rivero, Lara M. Hochfeld, Sugirthan Sivalingam, Markus M. Nöthen, Stefanie Heilmann-Heimbach

**Affiliations:** 1 Institute of Human Genetics, University of Bonn, School of Medicine & University Hospital Bonn, 53127 Bonn, Germany; 2 Present address: Department of Genetic Epidemiology, Institute of Human Genetics, University of Münster, 48149 Münster, Germany

**Keywords:** Hair follicle, Hair loss, Expression quantitative trait loci, Androgenetic alopecia, Single nucleotide polymorphisms

## Abstract

**Background:**

The association of molecular phenotypes, such as gene transcript levels, with human common genetic variation can help to improve our understanding of interindividual variability of tissue-specific gene regulation and its implications for disease.

**Methods:**

With the aim to capture the spectrum of biological processes affected by regulatory common genetic variants (minor allele frequency ≥ 1%) in healthy hair follicles (HFs) from scalp tissue, we performed a genome-wide mapping of *cis*-acting expression quantitative trait loci (eQTLs) in plucked HFs, and applied these eQTLs to help further explain genomic findings for hair-related traits.

**Results:**

We report 374 high-confidence eQTLs found in occipital scalp tissue, whose associated genes (eGenes) showed enrichments for metabolic, mitotic and immune processes, as well as responses to steroid hormones. We were able to replicate 68 of these associations in a smaller, independent dataset, in either frontal and/or occipital scalp tissue. Furthermore, we found three genomic regions overlapping reported genetic loci for hair shape and hair color. We found evidence to confirm the contributions of PADI3 to human variation in hair traits and suggest a novel potential candidate gene within known loci for androgenetic alopecia.

**Conclusions:**

Our study shows that an array of basic cellular functions relevant for hair growth are genetically regulated within the HF, and can be applied to aid the interpretation of interindividual variability on hair traits, as well as genetic findings for common hair disorders.

**Supplementary Information:**

The online version contains supplementary material available at 10.1186/s12895-020-00113-y.

## Background

Human hair traits show wide interindividual variability, which has been suggested to be largely determined by genetic factors [1, 2].

Systematic gene identification efforts for a growing number of quantitative and complex human traits have shown that the majority of associated genetic factors are located in non-coding genomic regions [3]. These variants most probably exert their functional effects through the tissue-specific modulation of the expression of trait-relevant genes. Expression quantitative trait loci (eQTL) analyses, that correlate sequence variation with gene expression data, have proven a valuable tool in terms of delineating the tissue-specific architecture(s) of gene regulation and predicting the impact that trait-associated variants exert on it [4]. This approach therefore bridges the gap between a genetic association finding and the underlying biological mechanism, and may provide crucial insights into disease development.

Increased knowledge of the genetic factors that contribute to variability of gene regulation in the human scalp hair follicle (HF) will aid the interpretation of genetic findings for hair-related traits and hair loss disorders, such as androgenetic alopecia (AGA). Moreover, the comparison of the regulatory architecture between HFs from different scalp areas may aid the understanding of the variable susceptibility of hair follicle subpopulations to hormonal hair loss. The aims of the present study were to: 1) perform a systematic mapping of eQTLs in the human hair follicle, and 2) evaluate the potential of these eQTLs in terms of the functional annotation of genetic loci that contribute to the development of hair-related traits and common diseases.

## Methods

### Sample collection

About 50 HFs were plucked from the occipital scalp of 100 (discovery sample), and the frontal and occipital scalp of 25 (replication sample, previously described in [5]) unrelated male volunteers. Peripheral venous blood samples were collected from all study participants. All volunteers were German residents of European descent, and showed a collective mean age of 27.9 years.

### Extraction of nucleic acids

DNA was extracted from whole blood samples using the Chemagic Magnetic Separation Module I (Perkin Elmer Chemagen Technology Inc., Baesweiler, Germany). Total RNA was extracted from HFs using the RNeasy Micro Kit (Qiagen, Hilden, Germany), and the quality and quantity of the RNA were assessed using a BioAnalyzer 2100 (Agilent Technologies, Waldbronn, Germany), and a NanoDrop ND-1000 spectrophotometer (Peqlab Biotechnologie, Erlangen, Germany), respectively. Only total RNA samples with an RNA integrity number (RIN) ≥ 8 were further analyzed in the study.

### Array hybridization

DNA extracts were hybridized onto the Human OmniExpress-12v1.0 bead array (Illumina, San Diego, CA, USA) (*N* = 100) or the Illumina PsychArray v1.0 (*N* = 25) for genome-wide genotyping, while RNA extracts from HFs were amplified and biotinylated using the TotalPrep™-96 RNA Amplification Kit (Illumina, San Diego, CA, USA) prior to whole transcriptome profiling, performed on the Illumina HT-12v4 bead array.

### Preparation of genotype data

SNP array raw data was initially analyzed using the Genotyping module within the GenomeStudio software (Illumina). Genotype calls were exported for basic quality control in PLINK v1.9 ([6]; www.cog-genomics.org/plink/1.9/) to eliminate bad quality data (e.g. SNPs and individuals with high degree of missing data, very rare SNPs) prior to genotype imputation. Imputation was performed on the Michigan Imputation Server [7] using the 1000 Genomes Project Phase 3 v5 reference panel and Eagle v2.3 phasing [8]. Post-imputation data processing was performed using VCFtools [9] and quality control was carried out in PLINK 1.9. Briefly, only biallelic single nucleotide variants with high imputation quality score (Rsq) > 0.7, minor allele frequency (MAF) ≥ 1% and under Hardy-Weinberg equilibrium (HWE *p* > 1 × 10^− 8^) were further analyzed. A principal component analysis (PCA) was performed with PLINK to identify potential outlier samples and use the generated principal components (PCs) as covariates for eQTL analysis. Two final autosomal genotype datasets consisted of 5,887,234 SNPs and 98 individuals for the discovery sample, and 1,044,566 SNPs and 24 individuals for the replication sample.

### Preparation of gene expression data

Raw data from the expression microarrays was initially analyzed using the Gene Expression module within the GenomeStudio software to generate calls and detection *p*-values. The probe-level gene expression data was exported for pre-processing by background correction, quantile normalization, log2 transformation, probe quality filtering and identification of potential outlier samples by PCA using R. Probes were considered expressed when showing a detection p-value < 0.01 in at least 5% of the samples. Probe quality filtering included the retention of only “good” and “perfect” quality probes mapping to only one gene with a valid identifier, according to annotations retrieved from the illuminaHumanv4.db package [10]. Three final gene expression datasets consisted of 13,217 probes and 98 individuals for the discovery sample, and 13,091 probes from frontal scalp and 12,814 probes from occipital scalp and 24 individuals for the replication sample.

### eQTL mapping

Genome-wide associations in *cis* (1 Mb window) between the expression levels in scalp HFs and SNP genotypes were tested using QTLtools [11]. Three covariate files prepared for the analyses included the first 10 (discovery sample) or 5 (replication sample) PCs for the genotype datasets and the first 10 or 5 PCs for the phenotype datasets. Initially, the full spectrum of eQTLs for each of all three gene expression datasets was identified through nominal pass analysis. After exploration of the nominal significant results (*p* < 0.05), only those eQTLs with false discovery rate (FDR) < 1 × 10^− 4^ in the discovery sample were considered true eQTLs, while all eQTLs with *p* < 0.01 in the replication sample were retained for further analyses. To identify independent signals within our set of true eQTLs, a permutation pass analysis (1000 permutations), followed by a conditional pass analysis on grouped phenotypes (i.e. a gene-level output from the probe-level analysis) were applied.

### Variant annotation

We explored reported eQTL effects for our independent HF eQTLs using the Variant Annotation tool from SNiPA (Single Nucleotide Polymorphisms Annotator) ([12]; http://snipa.org), noting whether or not the HF eQTL has been previously reported to have *cis*-eQTL effects in at least one tissue, and whether or not eQTL effects have been observed on the same gene as in our study. Additionally, we searched for trait associations of our true HF eQTLs that have been reported in the GWAS Catalog ([13]; https://www.ebi.ac.uk/gwas/).

### Replication of HF eQTLs

The true eQTLs identified from the nominal pass analysis in the discovery sample were considered replicated when the same eQTL SNP (eSNP) was found associated to the same eGene, either through the same or a different probe, with *p* < 0.01 in either frontal and/or occipital scalp areas from the replication sample.

### Differential eQTLs between frontal and occipital scalp

To investigate differential regulatory effects between frontal and occipital scalp areas, non-overlapping eQTLs with *p*-value < 5 × 10^− 5^ were investigated in the replication sample. These included eSNPs that showed different effects between both datasets (i.e. association to a different eGene or opposing direction of the effect for the same SNP-gene pair; *different-effect* eQTLs), and those eQTLs that were unique to the frontal or occipital dataset (i.e. no overlaps in eSNPs or eGenes; *region-specific* eQTLs).

### Functional enrichment analysis

The lists of eGenes obtained from the discovery sample and the differential eQTL analysis were submitted for enrichment analysis using the GENE2FUNC function of the Functional Mapping and Annotation of Genome-Wide Association Studies (FUMA GWAS) platform ([14]; http://fuma.ctglab.nl/). Each analysis used the provided option to include all human genes as background, a significance cutoff of FDR < 0.05 and a threshold of minimum overlapping genes with the gene-sets = 3. In addition, a comparative analysis of biological pathways for the differential eQTLs was performed using the lists of the identified region-specific eGenes and the Gene Enrichment Compare function within the FunRich (Functional Enrichment analysis tool) software [15]. From the resulting pathway analysis using the default FunRich database, only frontal- and occipital-specific terms with at least 2 genes from the dataset present in the pathway term, and *p* < 0.01 (from hypergeometric test) in one scalp area and *p* > 0.05 in the other, were retained for the purposes of the present study.

### Overlaps with reported genetic findings for hair phenotypes

To test the informativeness of our HF eQTLs for the interpretation of genetic findings for hair phenotypes, we used three sets of published genome-wide significant variants (*p* < 5 × 10^− 8^) associated with (i) hair shape (discovery meta-analysis, supplementary table 2 from [1]), (ii) hair color (meta-analysis, supplementary table 2 from [2]), and (iii) AGA (reported lead SNPs from [16–26]). These three variant sets were subjected to analysis by the SNP2GENE function of FUMA GWAS to assign GWAS findings to genomic regions. The analysis for each phenotype was set to include SNPs from the 1000 Genomes Project (Phase 3, European population) [27] that are in linkage disequilibrium (LD) with the reported GWAS variants. LD blocks were set to include variants of minor allele frequency ≥ 0.01, *r*^*2*^ ≥ 0.6 and a distance < 500 kb for merging into a locus. Afterwards, we searched for overlaps between our true HF eQTLs and the resulting loci for hair shape, hair color and AGA.

## Results

### Hair follicle eQTLs

**Fig. 1:**
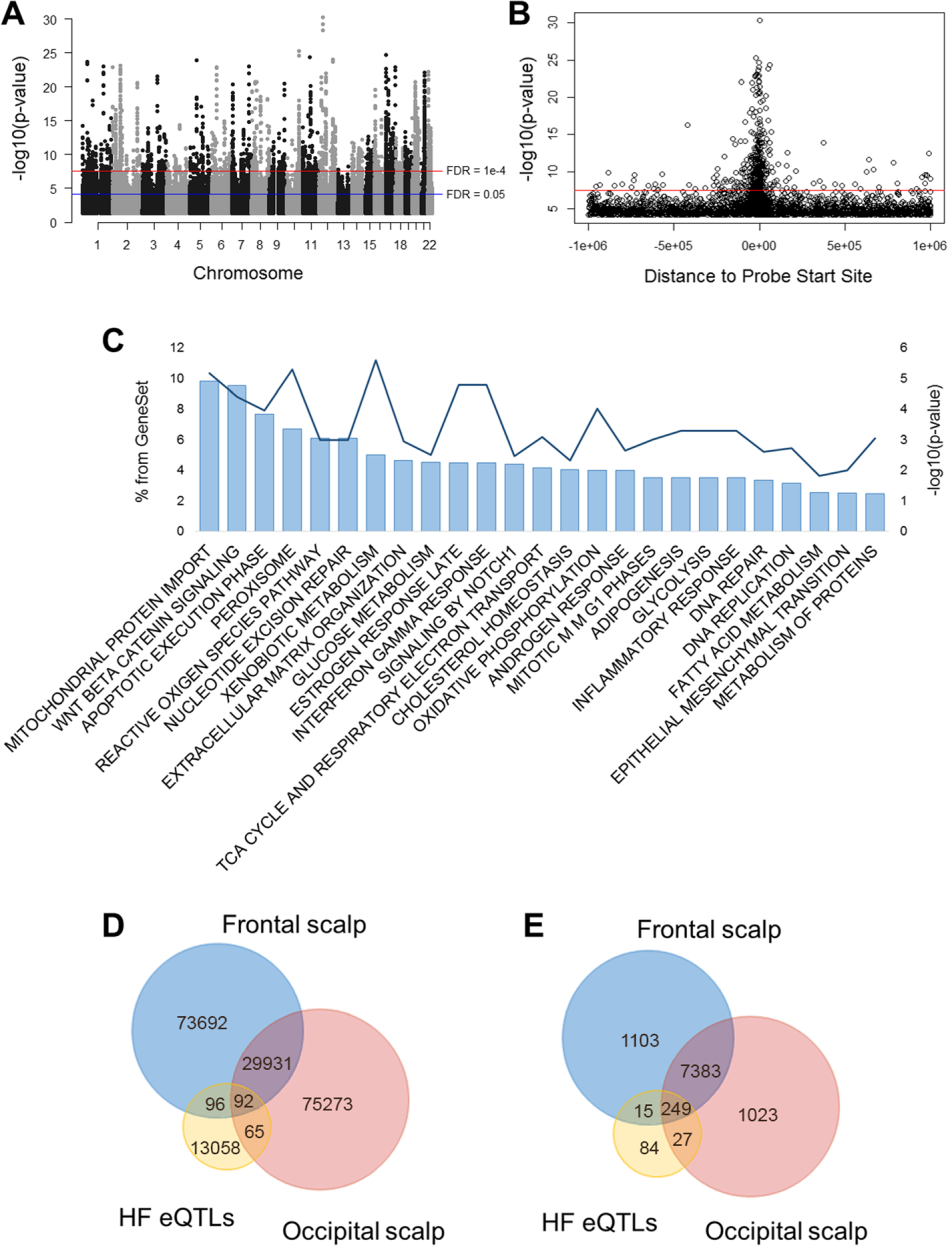
Hair follicle eQTLs*.* **a** Manhattan plot showing all nominally significant (*p* < 0.05) eQTLs. The blue and red lines represent cutoff thresholds at FDR = 0.05 and FDR = 1 × 10^− 4^, respectively. **b** Plot showing the *P*-value and distance to probe start site of all top eQTLs (best eSNP per probe) with FDR < 0.05. The blue line represents the cutoff threshold at FDR = 1 × 10^− 4^. **c** Representative pathways enriched for the list of eGenes of true eQTLs. **d** SNP overlaps between the true hair follicle eQTLs (FDR < 1 × 10^− 4^) and the relaxed frontal and occipital eQTLs (*p* < 0.01) datasets. **e** Gene overlaps between the true hair follicle eQTLs and the relaxed frontal and occipital eQTLs datasets

**Fig. 2:**
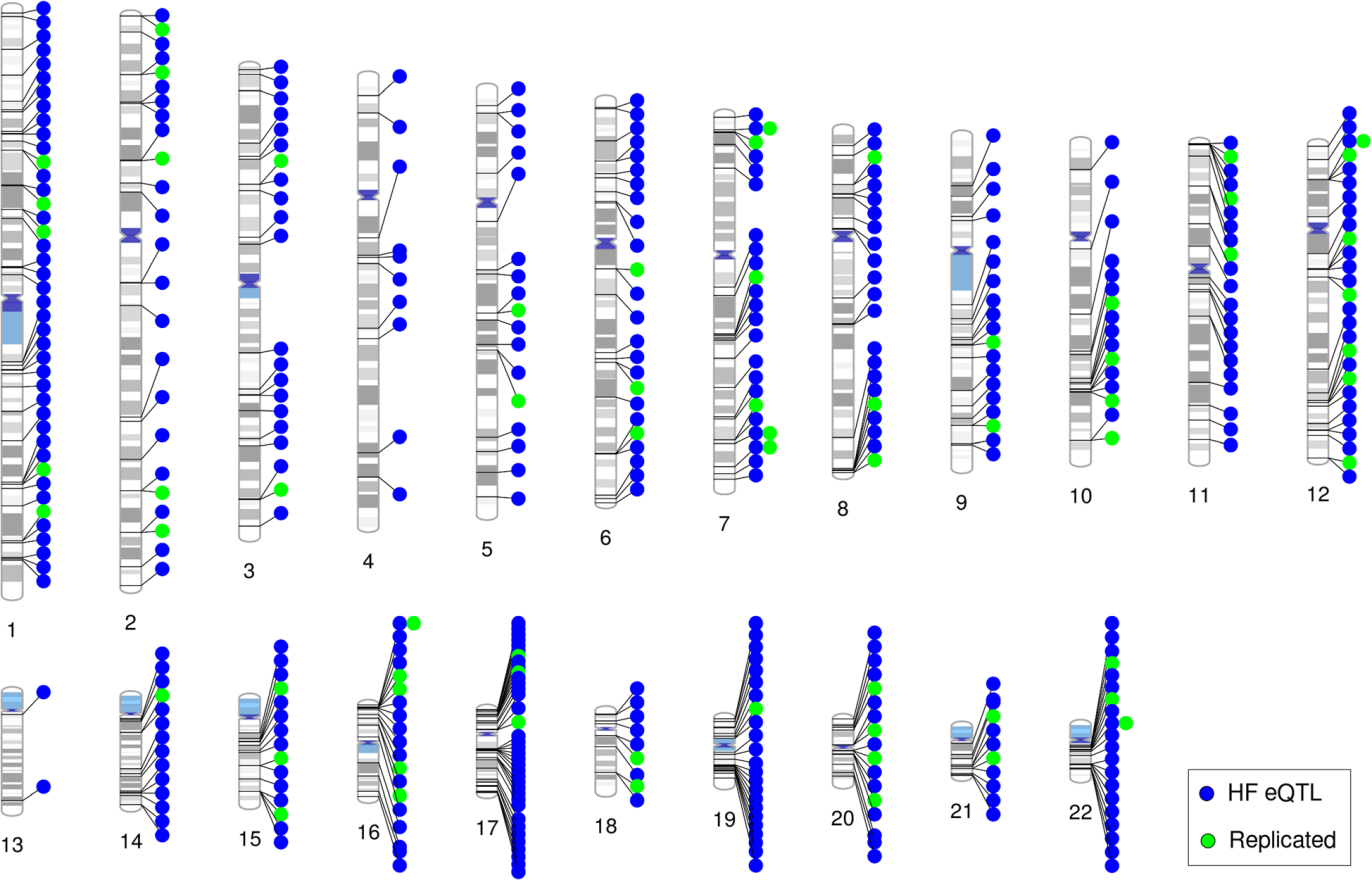
Overview of hair follicle eQTLs*.* Visualization of the chromosomal locations of our set of 374 independent HF eQTLs, as well as the 68 best replicated eQTLs (considered as independent signals)

We identified almost 3 million nominally significant eQTL signals in HFs from the occipital scalp (Fig. 1a). However, when we plotted the *p*-values and distances to the transcription start site (TSS) of the probe for each top SNP-probe pair with FDR < 0.05, we observed that a large number of SNPs were lying over 250 kb from the probe’s TSS, suggesting many of these might actually be noise signals (false positives). For this reason, only eQTL-findings that showed an FDR < 1 × 10^− 4^ were considered the likely true eQTLs (14,497 probe-SNP pairs) (Fig. 1b). From this set of true eQTLs, we identified a total of 374 independent HF eQTLs (Fig. 2a, Suppl.Table.1 [Supplementary Tables]). The top 20 independent eQTL findings are presented in Table 1. Functional enrichment analyses using FUMA revealed that associated eGenes were highly enriched (FDR < 0.05) for a variety of Hallmark and Reactome pathways related to metabolism, immune functions, cellular proliferation, apoptosis, adipogenesis and responses to sex hormones (Fig. 1c, Suppl.Table.2 [Supplementary Tables]). Additionally, the annotation of reported eQTL effects with SNiPA showed that 266 of 374 independent eSNPs have been previously reported to have *cis*-eQTL effects in at least one other tissue, from which 228 were reported to affect the same eGene. Annotation of GWAS Catalog trait associations identified 13 independent HF eSNPs that are associated with 15 traits, including type 2 diabetes mellitus and chronic inflammatory diseases. From the set of true HF eQTLs, 185 eSNPs showed associations with 134 traits, with the highest number of associations found for body mass index (associated with 29 eSNPs) and blood protein levels (associated with 13 eSNPs) (data not shown).

**Table 1:** Top findings for hair follicle eQTLs

Gene	# eQTLs	Best eSNP	Chr	Position	A1	A2	P	FDR	Effect
*Top 20 independent eQTLs (discovery sample)*									
*IPO8*	183	rs7326	12	30,782,301	G	A	5.17E-31	1.56E-24	−0.6840
*ATP5MD*	95	rs2271751	10	105,175,131	C	T	5.80E-26	3.21E-20	1.1004
*C17ORF97*	35	rs11150881	17	259,304	G	A	2.18E-25	1.18E-19	0.6600
*TIMM10*	9	rs3851117	11	57,237,113	A	G	4.60E-25	2.11E-19	−0.8391
*KCTD10*	211	rs4766601	12	109,890,080	C	G	9.89E-25	4.49E-19	0.5773
*C5ORF35/SETD9*	50	rs2591963	5	56,237,135	A	T	1.50E-24	6.43E-19	−0.6930
*NDUFS5*	45	rs374279960	1	39,484,742	G	T	2.36E-24	1.01E-18	−0.2496
*LOC339804*	267	rs3213944	2	61,372,298	G	C	8.36E-24	2.50E-18	0.4471
*FMO1*	45	rs6674596	1	171,235,088	T	A	1.04E-23	3.07E-18	1.3913
*PARP12*	32	rs2286197	7	139,726,467	G	A	1.19E-23	3.46E-18	0.3402
*SH3YL1*	50	rs62114506	2	242,793	C	G	1.30E-23	3.78E-18	−0.5886
*PEX6*	57	rs3805946	6	42,955,749	C	T	1.47E-23	4.11E-18	0.5651
*FN3KRP*	109	rs2249888	17	80,675,738	G	A	1.54E-23	4.11E-18	0.3341
*BCR*	44	rs131703	22	23,652,201	G	A	6.23E-23	1.36E-17	0.4445
*PSMG1*	295	rs35064900	21	40,555,492	G	A	7.92E-23	1.55E-17	−0.3917
*RPS26L*	47	rs773114	12	56,379,060	T	A	1.14E-22	2.12E-17	0.4386
*GOLGB1*	24	rs9968051	3	121,384,081	G	C	3.20E-22	5.56E-17	−0.6104
*ERP27*	182	rs12312821	12	15,077,527	T	A	4.48E-22	7.63E-17	−0.6385
*SLC47A2*	51	rs12451902	17	19,619,063	G	A	1.02E-21	1.65E-16	0.6855
*ABHD12*	517	rs2258728	20	25,276,343	A	G	1.91E-21	2.88E-16	0.2737
*Top 10 eQTLs in frontal scalp (replication sample)*									
*TDRD6*	14	rs552053483	6	46,870,456	C	A	5.32E-12	4.08E-06	0.4313
*CXCL1*	32	rs191105830	4	75,248,687	G	A	3.07E-09	7.16E-04	0.4703
*ZXDC*	12	rs552704609	3	126,534,497	A	C	4.70E-08	0.00871	0.3945
*COA4*	2	rs34214542	11	74,207,014	G	T	7.82E-08	0.01141	0.3317
*DLG2*	25	rs141374880	11	83,245,973	A	G	8.60E-08	0.01141	0.4126
*TADA2A*	3	rs74398459	17	36,095,246	G	T	1.17E-07	0.01446	0.3338
*C17orf67*	2	rs187188078	17	55,455,184	T	C	1.46E-07	0.01761	0.6551
*FAM220A*	16	rs188128204	7	6,373,651	A	G	2.11E-07	0.02083	0.4342
*ARMC6*	5	rs150042710	19	18,518,809	T	C	2.40E-07	0.02262	0.6410
*NUP153*	10	rs115808997	6	17,571,313	T	C	4.54E-07	0.03605	−0.4850
*Top 10 eQTLs in occipital scalp (replication sample)*									
*TELO2*	17	rs561125041	16	1,464,247	T	C	8.51E-10	3.00E-04	1.2115
*LPHN1*	17	rs147147550	19	14,373,177	T	C	9.90E-10	3.00E-04	0.6231
*ENTHD2*	5	rs117860115	17	78,992,225	T	C	4.10E-09	0.00121	0.1629
*GCSAM*	2	rs139726887	3	112,327,159	T	C	3.41E-08	0.00496	0.3622
*CCL2*	11	rs72825069	17	32,600,928	T	C	6.38E-08	0.00678	1.7221
*RUFY3*	3	rs115702490	4	72,452,170	T	C	1.24E-07	0.01296	0.4149
*GGPS1*	6	rs115335216	1	236,067,212	T	G	2.13E-07	0.01422	0.2215
*MTF1*	10	rs3935450	1	37,656,324	G	T	2.65E-07	0.01763	−0.2218
*CXADR*	4	rs55707799	21	18,849,240	C	T	3.47E-07	0.02132	−0.4272
*KRT37*	1	rs35490951	17	40,250,939	C	A	3.65E-07	0.02132	1.8106

The top 20 hair follicle eQTLs obtained from human occipital scalp in the discovery sample and the top 10 hair follicle eQTLs obtained from frontal and occipital scalp in the replication sample are shown

*eQTLs* expression quantitative trait loci, *eSNP* SNP with eQTL effect, *Chr* chromosome, *A1* effect allele, *A2* other allele, *P p*-value, *FDR* false discovery rate

We achieved the replication of 68 independent signals (Fig. 2) in either occipital and/or frontal scalp HFs from a total of 255 overlapping eQTLs (Suppl.Table.3 [Supplementary Tables]). In total, 188 and 157 eSNPs, and 264 and 276 eGenes, overlapped between our true eQTLs and the frontal and occipital HF eQTLs from the replication sample, respectively. Although only 92 eSNPs overlapped between all three eQTL datasets, these accounted for 249 eGenes (Fig. 1d and e); however, not all eQTL effects were consistent between the three datasets.

### Differential HF eQTLs between frontal and occipital scalp areas

We included all eQTLs with *p* < 5 × 10^− 5^ in the analysis of the replication sample. However, as the power to detect associations is markedly reduced due to the limited sample size (*N* = 24), this is reflected in the number and statistical significance of eQTLs that we detected in frontal and occipital scalp, as well as in their distributions with respect to the probe’s TSS (Suppl.Figure.1 [Supplementary Figures]). In general, we found little overlap between these two datasets, and will limit ourselves here to briefly present an overview of regional differences between frontal and occipital scalp.

We identified 71 HF eQTLs with inconsistent effects between frontal and occipital HFs. These were considered “different-effect eQTLs” and affected 11 genes (Suppl.Table.4 [Supplementary Tables]). Furthermore, we identified 289 frontal (Suppl.Table.5 [Supplementary Tables]) and 339 (Suppl.Table.6 [Supplementary Tables]) occipital (region-specific) eQTLs, from which the top 10 for each scalp region are shown in Table 1. To identify differentially-enriched pathways, we performed a separate FUMA pathway analysis for frontal and occipital differential eGenes (i.e. region-specific + different-effect) and compared the results for both scalp regions. This analysis suggested differences in the genetically-determined regulation of the responses to steroid hormones, cell cycle control, cellular metabolism and immune functions between the scalp regions (Suppl. Figure.2 [Supplementary figures]). However, as several of the region-specific pathway terms seemed redundant, we sought to further elucidate the differences between both scalp regions through a comparative pathway analysis using FunRich. This analysis suggested that the more important differences between frontal and occipital scalp HFs are on pathways related to the metabolism of amino acids and signaling by histone deacetylases (HDACs) in the frontal scalp, and to proliferative processes in the occipital area (Table 2).

**Table 2:** Best frontal- and occipital-specific biological pathways enriched for differential eGenes

Biological pathway	GeneSet	Frontal scalp			Occipital scalp		
		n	Fold value	P	n	Fold value	P
*Frontal scalp eQTLs*							
Activation of Chaperones by ATF6-alpha	9	3	21.85	2.78E-04	0	0.01	1
Interconversion of 2-oxoglutarate and 2-hydroxyglutarate	3	2	43.68	6.92E-04	0	0.01	1
Pyruvate metabolism and Citric Acid (TCA) cycle	31	4	8.46	1.22E-03	0	0.01	1
Arginine degradation I (arginase pathway)	4	2	32.78	1.37E-03	0	0.01	1
Proline biosynthesis II (from arginine)	5	2	26.24	2.26E-03	0	0.01	1
Arginine degradation VI (arginase 2 pathway)	6	2	21.87	3.36E-03	0	0.01	1
Vitamin C (ascorbate) metabolism	6	2	21.87	3.36E-03	0	0.01	1
Dolichyl-diphosphooligosaccharide biosynthesis	7	2	18.75	4.65E-03	0	0.01	1
Signaling events mediated by HDAC Class I	113	6	3.48	7.84E-03	1	0.51	8.66E-01
Signaling events mediated by HDAC Class II	38	4	6.90	2.62E-03	1	1.51	4.91E-01
BMP receptor signaling	226	9	2.61	8.19E-03	5	1.26	3.66E-01
Amino acid synthesis and interconversion (transamination)	10	3	19.67	3.92E-04	1	5.74	1.63E-01
*Occipital scalp eQTLs*							
BARD1 signaling events	29	1	2.28	3.61E-01	4	7.86	1.57E-03
G0 and Early G1	21	0	0.01	1	3	8.15	5.66E-03
TGFBR	125	4	2.10	1.25E-01	7	3.19	6.64E-03
VEGF and VEGFR signaling network	1301	27	1.36	6.57E-02	35	1.53	8.04E-03

*eQTLs* expression quantitative trait loci, *GeneSet* number of total genes in pathway term, *n* number of genes from the study overlapping the pathway term, *P p*-vlaue

### Overlaps with genomic regions associated with hair phenotypes

**Fig. 3:**
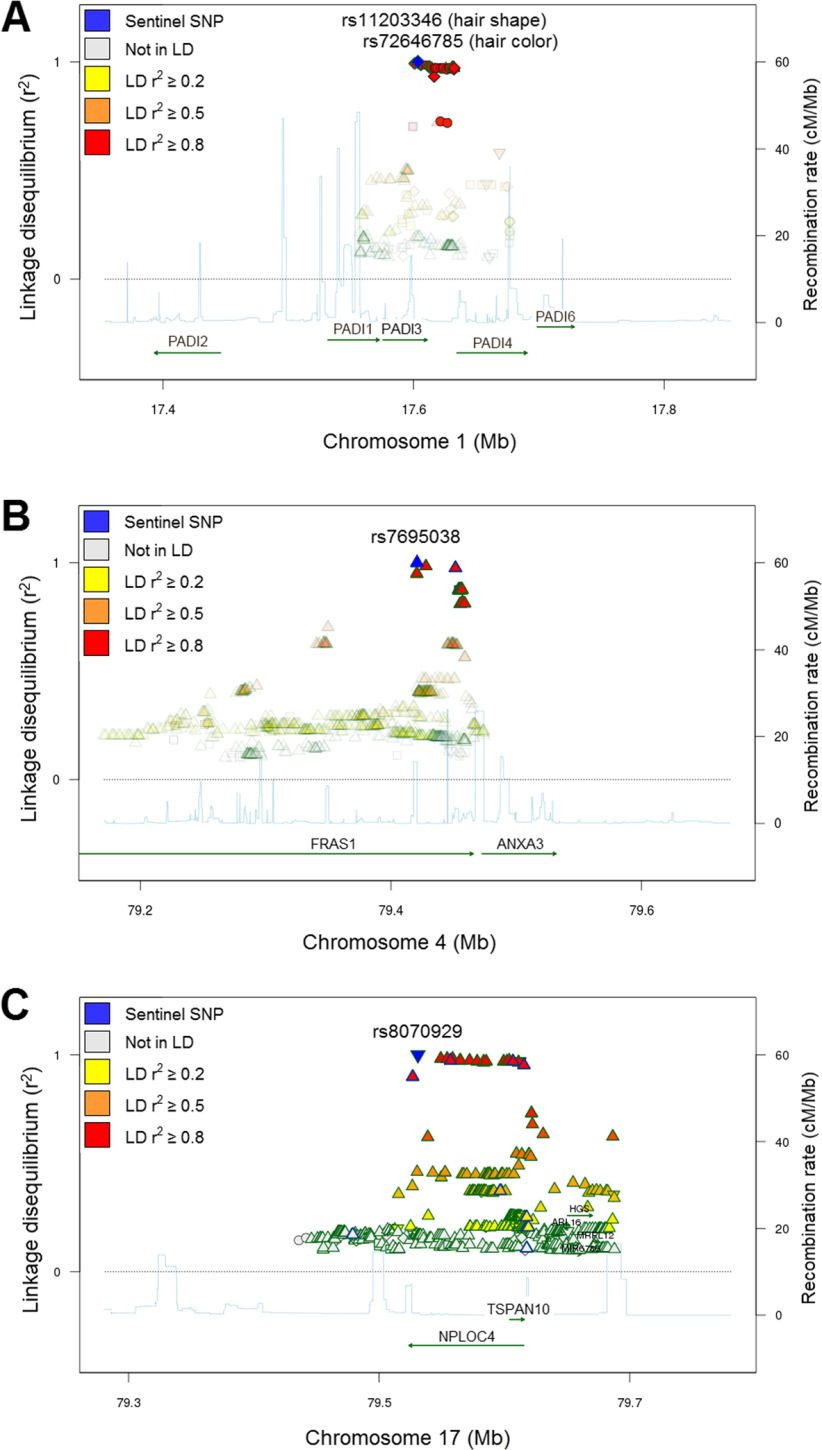
Hair follicle eQTLs overlapping genetic findings for hair-related traits*.* Linkage disequilibrium plots highlighting: **a** 31 HF eQTLs that overlapped a genomic locus shared by hair shape and hair color, **b** 26 HF eQTLs that overlapped a genomic locus for hair shape, with the most strongly associated SNP used as sentinel (in blue), and **c** one HF eQTL (blue) that overlapped a genomic locus for hair color. GWAS loci were defined through an analysis with the FUMA SNP2GENE function, applied separately to the genome-wide findings of GWAS for hair shape and hair color. The plots were generated using SNiPA. For the plot in **a**, rs72646785 (independent HF eQTL for the region) was used as the sentinel SNP (blue). The lead SNPs within this locus for hair shape (rs11203346) and hair color (rs72646785) are separated from each other by 2650 bp and have an LD *r*^*2*^ = 0.99 in the 1000 Genomes Project, Phase 3 v5, European population reference panel

To illustrate the applicability of our set of true eQTLs to interpret GWAS findings for hair phenotypes, we investigated for a potential overlap of HF eSNPs with genomic risk loci for AGA (98 loci), hair shape (12 loci) and hair color (122 loci) (Table 3). We identified one genomic locus at 1p36.13 that had previously been associated with hair phenotypes. This comprised 31 eSNPs overlapping association signals for hair shape (overlapping lead SNP: rs11203346) and hair color (overlapping lead SNP: rs72646785) (Fig. 3a). For this locus, the nearest genes were mapped to *PADI3* and *PADI4* in both GWAS, while the eGene for this region corresponded to *PADI3*. Moreover, another locus at 4q21.21, comprised by 26 eSNPs linked to the signal of rs6533756 (a non-eSNP in LD *r*^*2*^ ≥ 0.8 with all eSNPs), was associated with hair shape. The overlapping eSNP most strongly associated with hair shape at this locus was rs7695038 (*p* = 3.9 × 10^− 10^). While eSNPs at this locus locate within a region coding for the *FRAS1* gene, the associated eGene, *ANXA3,* was located 66,182 bp downstream of *FRAS1* (Fig. 3b). A third locus at 17q25.3 comprised the eSNP, rs8070929, which was in LD (*r*^*2*^ ≥ 0.97) with rs34872037 (a non-eSNP) that is associated with hair color. While the nearest gene for the SNP at this locus was *NPLOC4*, the HF eGene corresponded to *TSPAN10* (Fig. 3c). All eSNPs overlapping association signals for hair shape and/or hair color can be found in Suppl.Table.7. We found no overlaps of our true HF eQTL loci with the reported genome-wide significant findings for AGA. However, we observed that the eGene *ATP2B4* was annotated as the nearest gene to an AGA genomic locus at 1q32.1. Moreover, it is perhaps worth to notice that, at the nominal significance level (*p* < 0.05), we found 93 eGenes to overlap with reported AGA candidate genes, of which 18 were associated with eSNPs at an FDR < 0.05 (*ALPL, B3GNT8, BCL2, DIP2B, FAM136A, HDAC9, LCLAT1, LHPP, NSF, PRRX1, RPTN, RSPO2, SUCNR1, TCHH, TMEM50A, TWIST2, MSIG2 and SPAG17*).

**Table 3:** Genomic loci for hair phenotypes and their observed overlaps with our hair follicle eQTLs

Summary	Hair shape	Hair color	AGA
Sample size	16,763	290,891	145,000^a^
# Genomic loci	12	122	98
# Lead SNPs	23	124	185
# Ind.Sig.SNPs	63	130	245
# Candidate SNPs	1424	6658	11,821
# GWAS SNPs	704	134	335
# Mapped genes	69	204	161
# Total input SNPs	706	137	387
# Skipped input SNPs	2	3	36
Overlapping eGenes with mapped genes	*PADI3*	*BCAS1, C17orf70, CHMP4C, MRPL39, PADI3, RYBP*	*ATP2B4*
Overlapping loci	2	2	–
Overlapping Chr.Loc.	chr1p36.13	chr1p36.13	–
	chr4q21.21	chr17q25.3	
Mapped genes	*PADI3, PADI4*	*PADI3, PADI4*	–
	*FRAS1*	*NPLOC4*	
eGenes	*PADI3*	*PADI3*	–
	*ANXA3*	*TSPAN10*	

*AGA* androgenetic alopecia, *Ind.Sig.SNPs* independent significant SNPs, *Chr.Loc.* chromosome location

^a^Approximated sample size, as lead SNPs were collected from different studies

## Discussion

The strongest eQTL associations in occipital HFs were observed for *IPO8* (rs7326), *ATP5MD* (rs2271751), and C17orf97/LIAT1 (rs11150881). The findings for *ATP5MD* were confirmed in our small replication study. Although we were not able to replicate the “true” SNP-gene associations for *IPO8* in our replication sample, a set of different SNPs were indeed associated with *IPO8* expression in HFs from frontal and/or occipital scalp areas, some of which were also associated with *IPO8* expression in the discovery sample at the nominal level (data not shown), therefore confirming the genetic regulation of *IPO8* in HFs. While little is known about the function of C17orf97, IPO8 mediates the nuclear import of proteins and mature microRNAs [28]. ATP5MD is crucial for the maintenance of ATP synthase in mitochondria, and might actively participate in the cellular energy metabolism, a process with well-known relevance to hair biology and hair growth [29, 30]. Over-expression of *ATP5MD* causes a number of mitochondrial abnormalities and an increase in anaerobic metabolism associated with the induction of an epithelial to mesenchymal-like transition, as well as delayed cell growth [31, 32]. The genetically-controlled regulation of mitochondrial function in human HF is further supported by our pathway analysis that found an enrichment of eGenes for true eQTLs in pathways related to mitochondrial function along with other pathways, such as the regulation of responses to steroid hormones, the Wnt/β-catenin and interferon (IFN) signaling, adipogenesis, immune responses and the metabolism of glucose and lipids, all of which have well-known roles in HF biology [33].

We also investigated whether there might be differences in the genetic control of gene expression between HF subpopulations from different scalp areas. Despite the reduced size of this (replication) sample, our results point to interesting avenues for future research. For instance, an important difference between frontal and occipital scalp appears to be the metabolism of amino acids, including arginine degradation. It is known that the HF is dependent on arginine, as hair growth depends on the vasculature and L-arginine not only participates in cell proliferation but is a precursor for the vascular mediator nitric oxide. L-arginine deficiency has been shown to impair hair elongation, while its supplementation increases the number of HFs in anagen (growth) and decreases that of HFs in telogen (resting) phase [33, 34]. This supports the notion that regional increases in arginine degradation might result in enhanced vulnerability to hair loss in the frontal scalp area. A similar scenario can be thought for HDACs. HDACs are important transcriptional repressors that act in multiprotein complexes and are involved in the control of cell cycle progression [35, 36]. While class I HDACs are ubiquitously expressed, class II HDACs show tissue specificity [36]. In our study, we found eQTLs for HDACs 2, 5 and 7 only in frontal scalp. Interestingly, these particular HDACs modulate HF development and homeostasis [37, 38], as well as angiogenesis and vascular integrity [39–41]. Moreover, androgen actions have been shown to regulate HDAC7 subcellular compartmentalization, and HDAC7 has been proposed to be a co-repressor of the androgen receptor [42].

We also found evidence for differential regulation of vitamin C (L-ascorbic acid) metabolism in frontal scalp. It has been shown that a derivative of L-ascorbic acid (L-ascorbic acid 2-phosphate) promotes HF growth that is mediated by the induced expression of insulin-like growth factor-1 (IGF-1) in dermal papilla cells [43]. This opens the possibility that decreased expression of genes involved in the metabolism of L-ascorbic acid in frontal scalp might render this region more susceptible to hair loss. Taken together, our pathway analysis results show that frontal-specific pathways present several factors with negative effects on hair growth (e.g. androgen and estrogen responses, BMP2/4 signaling, IFN-γ response, endocannabinoid signaling), while occipital-specific pathways are more consistent with factors exerting positive effects on hair growth (e.g. vascular endothelial growth factor signaling, transforming growth factor beta receptor), considering what Bernard has referred to as “the Yin Yang of the human hair follicle” [33]. Nevertheless, the implications of genetic regulation in frontal scalp for hair loss disorders should be investigated through the generation of a confident eQTL dataset with increased power in future studies.

With our study, we also show that tissue-specific eQTL data are a valuable resource to identify regulatory effects at disease- and trait-associated loci. In particular, our results suggest an important role of PADI3 in hair traits. Indeed, PADI3 is located in the inner root sheath and medulla in anagen HFs and has been reported to play roles in HF differentiation [44] and hair shaft formation [45]. Our results also implicate *ANXA3* and *TSPAN10* as novel candidate genes for hair shape and color, respectively. Although we found no overlap between the true HF eQTLs and AGA genetic risk loci, which might be an expected finding, considering that the occipital scalp is not susceptible to balding, the overlapping eGene *ATP2B4* provides a potential novel candidate gene for AGA, as the reported gene for the region is so far *SOX13* [25, 26].

The most obvious limitation of our study is the sample size. However, we tried to overcome this limitation by applying high quality standards to the data and stringent selection criteria to our HF eQTL results. Another potential limitation of our study resides in the use of plucked HFs instead of intact HFs. However, a skin biopsy is required in order to obtain intact HFs, whereas hair plucking is a less invasive technique. Moreover, it has been demonstrated that plucked hairs retain most epithelial structures, maintain the integrity of the outer root sheet and also contain stem cells [46]. Finally, due to the small size of our samples, particularly that of the replication sample, we considered advisable to exclude from the present study indels and sex chromosomes, and limit ourselves to reporting, in very general terms, differential findings between HFs from frontal and occipital scalp areas.

## Conclusions

Our analyses demonstrate that well-established HF molecular pathways are genetically regulated and that, to some extent, this regulation can show regional specificity within scalp HFs. The enriched pathways mainly underscored processes that relate to hair growth and the HF cycle. More detailed tissue-specific analyses will be enabled by future increases in sample size, ensuring an improved understanding of the genetically determined variability in HF gene expression and its implications for hair-related traits and hair loss disorders.

## Supplementary Information

**Additional file 1.** Supplementary Tables.

**Additional file 2.** Supplementary Figures.

## Data Availability

The datasets used for this study are available from the corresponding author on reasonable request. The derived data supporting the conclusions of this article are included within the article and its additional files.

## Abbreviations

AGA: Androgenetic alopecia
eQTLs: Expression quantitative trait loci
FDR: False discovery rate
FUMA: Functional mapping and annotation of GWAS
GWAS: Genome-wide association analysis
HF(s): Hair follicle(s)
HDACs: Histone deacetylases
IFN: Interferon
LD: Linkage disequilibrium
MAF: Minor allele frequency
PCA: Principal component analysis
PCs: Principal components
RIN: RNA integrity number
SNP: Single nucleotide polymorphism
TSS: Transcription start site
