# Supplementary material for: Mapping of cis-acting expression quantitative trait loci in human scalp hair follicles
Source: BMC Dermatol. 2020 Nov 10;20:16. doi: 10.1186/s12895-020-00113-y (PMC7653834; doi:10.1186/s12895-020-00113-y)
Supplement: Supplementary file 1 — Additional file 1. Supplementary Tables. [file 12895_2020_113_MOESM1_ESM.pdf]

## SUPPLEMENTARY FIGURES

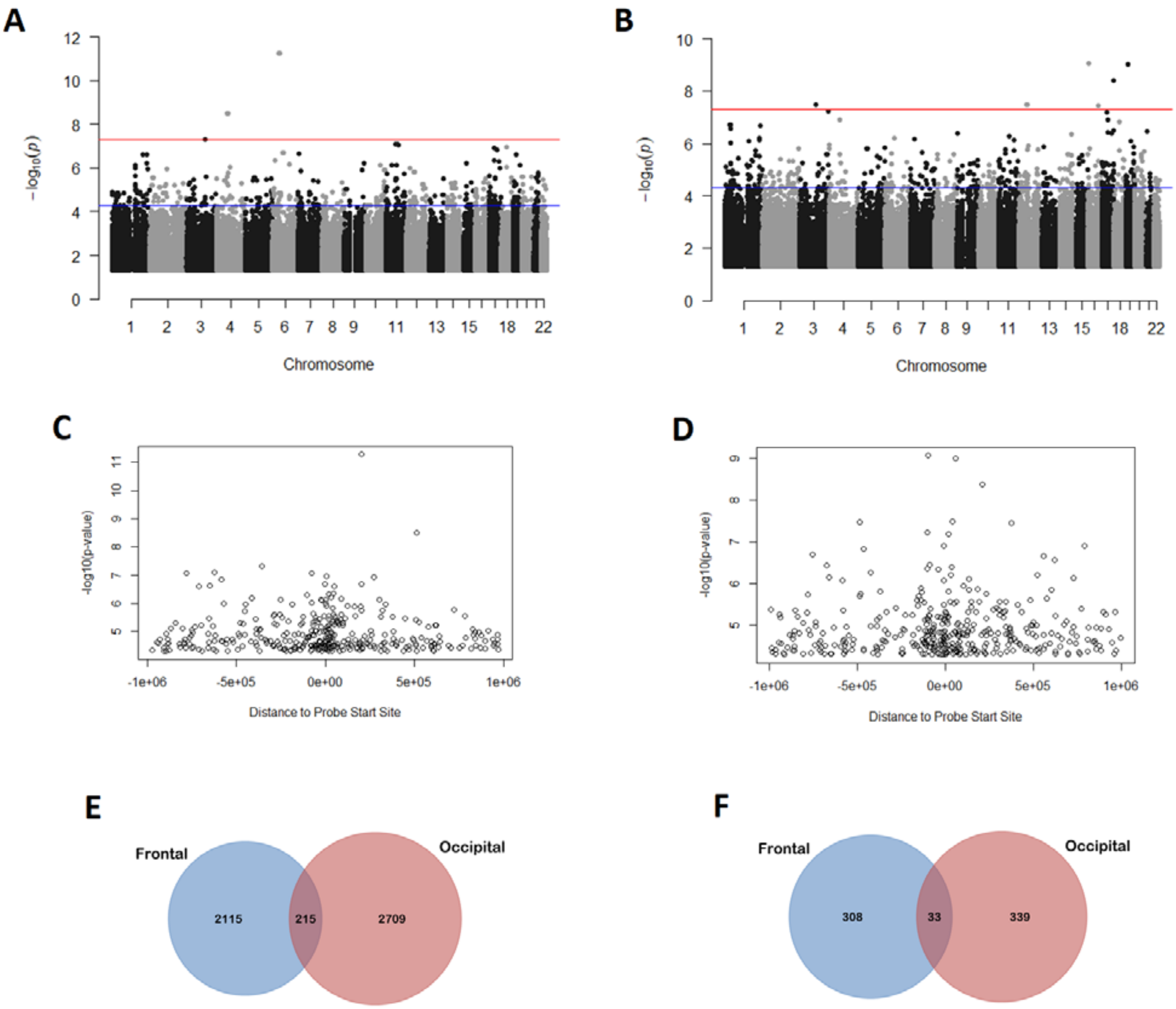


**Suppl.Figure.1.** Frontal and occipital scalp eQTLs in the replication sample. A) Manhattan plot of nominal frontal eQTLs. B) Manhattan plot of nominal occipital eQTLs. C) Plot of P-value and distance to probe start site for frontal eQTLs with $p < 5x10^{-5}$ (top variant per probe). E) Plot of P-value and distance to probe start site for occipital eQTLs with $p < 5x10^{-5}$ (top variant per probe). F) Venn diagram of eSNPs in frontal and occipital scalp. G) Venn diagram of eGenes in frontal and occipital scalp.

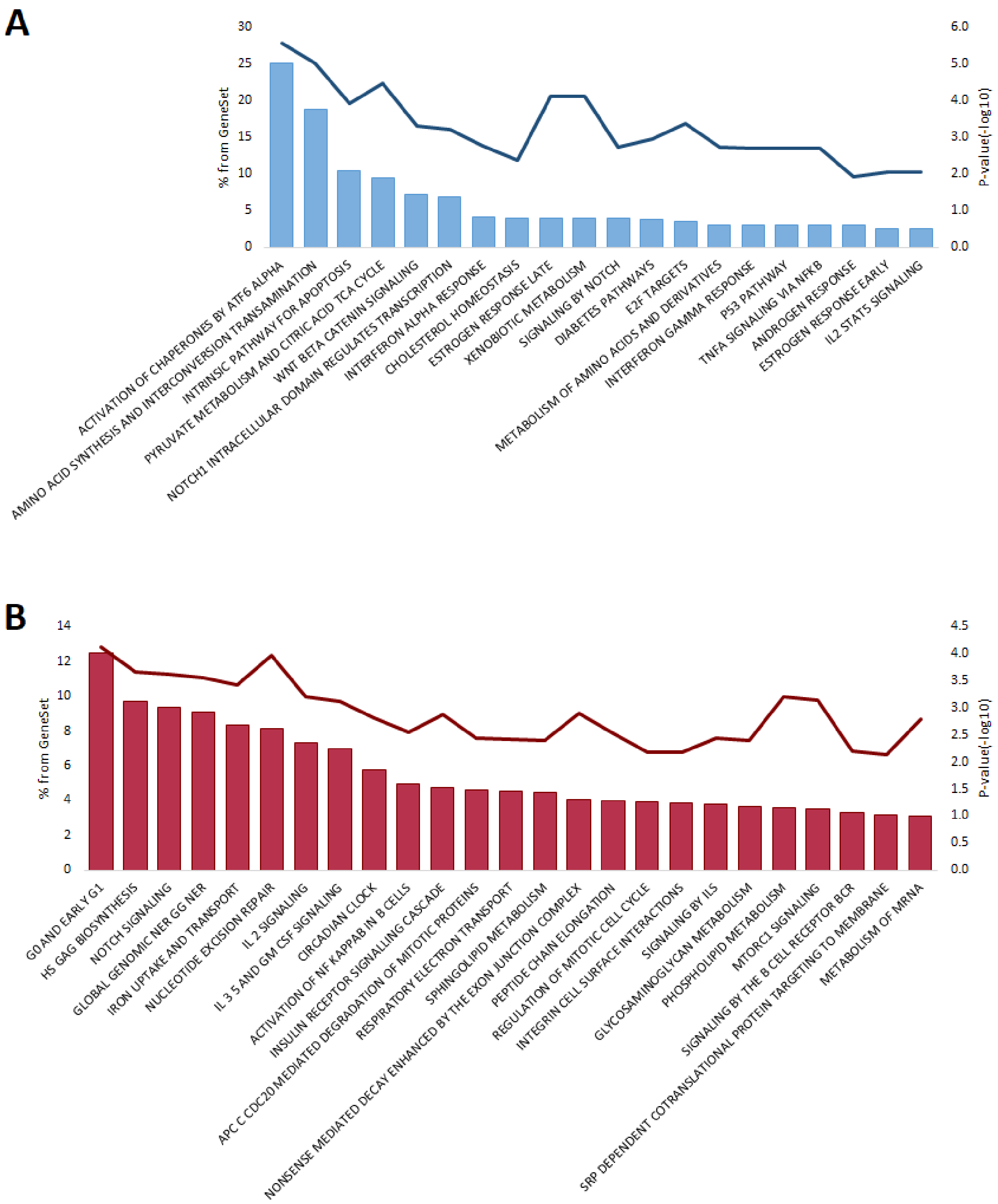


**Suppl.Figure.2.** FUMA Hallmark and Reactome pathway enrichments for the differential analysis.

A) Frontal-specific pathway terms. B) Occipital-specific pathway terms.
